# Characteristics of Preplaced Aggregate Concrete Fabricated with Alkali-Activated Slag/Fly Ash Cements

**DOI:** 10.3390/ma14030591

**Published:** 2021-01-27

**Authors:** Salman Siddique, Hyeju Kim, Hyemin Son, Jeong Gook Jang

**Affiliations:** Division of Architecture and Urban Design, Institute of Urban Science, Incheon National University, 119 Academy-ro, Yeonsu-gu, Incheon 22012, Korea; salmansiddique@inu.ac.kr (S.S.); hyeju_kim@inu.ac.kr (H.K.); sonhyemin@naver.com (H.S.)

**Keywords:** alkali-activated cement, preplaced concrete, filling capacity, total voids, compressive strength

## Abstract

This study assesses the characteristics of preplaced aggregate concrete prepared with alkali-activated cement grout as an adhesive binder. Various binary blends of slag and fly ash without fine aggregate as a filler material were considered along with different solution-to-solid ratios. The properties of fresh and hardened grout along with the properties of hardened preplaced concrete were investigated, as were the compressive strength, ultrasonic pulse velocity, density, water absorption and total voids of the preplaced concrete. The results indicated that alkali-activated cement grout has better flowability characteristics and compressive strength than conventional cement grout. As a result, the mechanical performance of the preplaced aggregate concrete was significantly improved. The results pertaining to the water absorption and porosity revealed that the alkali-activated preplaced aggregate concrete is more resistant to water permeation. The filling capacity based on the ultrasonic pulse velocity value is discussed to comment on the wrapping ability of alkali-activated cement grout.

## 1. Introduction

Alkali-activated binders can be obtained by the activation of raw materials in powder form under the action of soluble alkali silicates [1]. The materials in powder form are usually industrial by-products rich in silica and aluminum, such as fly ash [2], ground granulated blast furnace slag (GGBFS) [3], and metakaolin [4]. The alkali activation process consists of three distinct stages: The enrichment of the medium by the dissolution of aluminosilicates by reactive precursors during the reaction, restructuring by polycondensation to achieve a stable state of the precursors with the subsequent development of a gel phase, and then the final stage of gel hardening [5]. Precise details of the alkali activation process vary due to variations in the chemical compositions of the raw materials, the environmental conditions and the activation process used.

The development of alkali-activated binders as compared to traditional binders based on Portland cement has provided an environmental benefit with a comparatively low carbon footprint and lower processing energy requirements. Furthermore, the upcycling of industrial by-products provides an added advantage in that it is a resource-friendly process. Class F fly ash and GGBFS, given their aluminate and silicate contents, are generally considered as raw materials suitable for use as precursors in the development of alkali-activated materials [6]. Alkali-activated binders have been widely studied as part of the effort to develop better concrete and mortar systems [7], though few studies have focused on the development of alkali-activated grouts for use in construction applications. Idrissi et al. developed a geopolymer grout to be used in soil reinforcement applications [8]. They reported that combining different precursors and optimizing the water content are necessary to produce a rheological alkali-activated grout [8]. Aboulayt et al. developed a binary geopolymer grout consisting of metakaolin and fly ash [9]. They reported that the fly ash reduced the viscosity and increased the setting time [9]. Furthermore, the incorporation of metakaolin provided better homogeneity and flowability to the grout [9]. They concluded that the flowability of alkali-activated grout is an important parameter which is mainly influenced by particle properties such as their chemical compositions [9]. Idrissi et al. studied the resistance of alkali-activated grouts (binary blends of slag/metakaolin and fly ash) against acid leaching [10], reporting that alkali-activated slag/fly ash grout exhibited less volume loss as compared to metakaolin/fly ash grout [10]. The major cause of the superior performance of the alkali-activated slag/fly ash grout was the formation of a silica-rich gel [10]. Gullu et al. studied the rheology of a geopolymer grout based on fly ash and silica fume [11]. They concluded that up to 30% of fly ash and 40% of silica fume can be used to develop a geopolymer grout with suitable flow and rheological properties [11]. Idrissi et al. studied the chemical structure and long-term compressive strength of an alkali-activated grout based on slag and metakaolin (with and without fly ash) [12]. They reported that metakaolin-based grouts present a stable chemical structure, whereas slag-based grout presents an evolving chemical system with a negative impact on the compressive strength [12].

Preplaced concrete, also known as two-stage concrete or prepacked concrete, is a special type of concrete which is used in repair work and in large-scale concrete structures and structural elements with dense steel reinforcements [13]. Preplaced concrete is produced by placing coarse aggregates in the formwork and injecting a suitable type of grout to act as a binder. This fabrication technique is beneficial as it allows the use of large aggregates at any amount without concern over segregation. For grout injections, Portland cement has been used extensively; however, as pointed out by Li et al. [14], the lower compressive strength of the grout in this case usually leads to poor mechanical performance of prepacked concrete. To mitigate this shortcoming, Li et al. developed ultra-high-performance cement grout to fabricate two-stage concrete with coarse aggregates 40 mm in size [14]. It should be noted that Portland cement grout for use in preplaced concrete systems is developed using cement as a binder and sand as a filler [14,15] or is developed via a suitable coating material, such as polyurethane [16]. In contrast, in grouts based on an alkali-activated binder, no additional filler materials are considered [8,9,10,12]. In summary, the use of an alkali-activated grout in the development of preplaced concrete offers the potential to develop a novel construction material with a smaller carbon footprint with less consumption of natural resources while also exhibiting the desired mechanical properties.

The present study aims to develop a preplaced concrete fabricated via an injection of alkali-activated cement grout. The alkali-activated grout used here was designed by incorporating different ratios of fly ash to slag and varying the alkali-activating solution content. The properties of the grout were examined according to the flowability and compressive strength. The mechanical properties of preplaced alkali-activated concrete (PAAC) were evaluated in terms of the compressive strength and the ultrasonic pulse velocity (UPV). The UPV was also employed to estimate the filling capacity of the alkali-activated grout at different specimen depths. Furthermore, the density, water absorption and total voids were examined.

## 2. Experimental Program

### 2.1. Materials

Slag and type F fly ash obtained from local industries in South Korea were used as precursors to develop the alkali-activated grout used in this study. Ordinary Portland cement was used to produce the conventional cement grout. The Portland cement consisted of 21% SiO_2_, 62.5% CaO, 5.9% Al_2_O_3_, 3.2% Fe_2_O_3_, and 2.1% SO_3_. Table 1 presents the chemical compositions of the fly ash and slag. In this case, a 5M NaOH solution and Na_2_SiO_3_ (SiO_2_ = 29% wt, Na_2_O = 10% wt and H_2_O = 61% wt) were used to produce the alkali-activating solution. The mass ratio between the NaOH solution and the Na_2_SiO_3_ was maintained at 1:1 throughout the study. The alkali-activating solution was prepared 24 h before casting and was cooled under laboratory conditions. The design proportioning of alkali activating solution was considered by referring to the previous works of the authors [17,18]. The crushed stone used in this study are obtained from rock quarries and are normally used coarse aggregates for traditional concrete. The crushed stone has irregular and angular shape with rough texture. A uniform size of 19–26.5 mm was used under a surface-saturated condition. The specific size range was selected to ensure smooth penetration of the alkali-activated grout into the mold. The maximum size held to less than one-quarter of the minimum dimension of the cylindrical mold (100 mm). To ensure this, the coarse aggregate was sifted through sieves between 19.5 mm and 26.5 mm in size.

### 2.2. Sample Preparation

A special construction technique is usually considered to prepare preplaced concrete, meaning that the normal procedures of designing a mix for the calculation of the amount of grout were not applicable. Therefore, an experimental procedure was followed here to determine the amount of alkali activated grout. In step 1, a cylindrical mold (100 mm diameter and 200 mm height) was packed with coarse aggregate (saturated surface dry (SSD) condition), with water used to fill the spaces between the aggregate materials. To prevent any water leakage, a water sealant was applied around the surface of the mold. The water sealant was applied on the mold for 24 h before step 1. In step 2, the coarse aggregate materials were removed carefully and the volume of water remaining in the mold was measured. The same procedure was repeated ten times to determine the average volume of water. This volume of water was considered when determining the amount of alkali-activated grout required to produce the preplaced concrete. The details of the design mix for the grout are presented in Table 2. In this study, a Portland cement grout was designed to act as a reference. The water-to-cement ratio was kept constant at 0.5 for the Portland cement grout. For the alkali-activated grout, two different series were considered. In series 1, the mass ratio of fly ash in the slag-based binder was varied at 10%, 30% and 50% and solution-to-solid mass ratio was kept constant at 0.7. In series 2, the amount of fly ash and slag used were 30% and 70%, respectively, and the solution-to-solid ratio was varied at 0.6, 0.7, 0.8, and 0.9. The nomenclature followed in present study is as follows: CC represents the Portland cement mix; series 1 mixes are represented by FnSm, where Fn is amount of fly ash and Sm denotes the amount of slag; series 2 mixes are represented by FSx, where x represents the solution-to-solid ratio.

**Figure 1:**
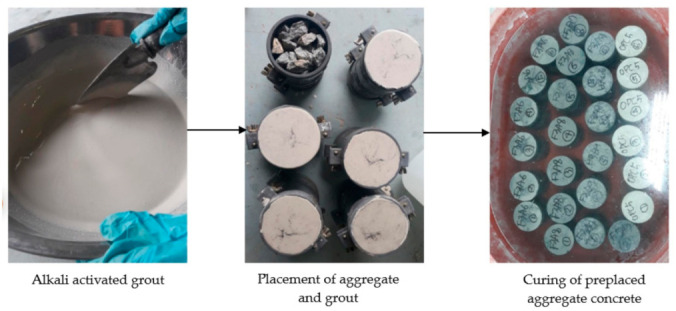
Fabrication of preplaced aggregate concrete specimen.

The SSD condition coarse aggregates were initially placed into a cylindrical mold with a diameter of 100 mm and height of 200 mm. The alkali-activated grout was prepared by dry mixing the solid precursors for one minute. The required alkali-activating solution was then added and mixed for three minutes to obtain a homogenous mix. The freshly prepared alkali-activated grout was then poured into the mold containing the preplaced coarse aggregates. A gravity-based grout injection was considered in the present study due to its convenience in casting and its economic benefit. The casting process is shown in Figure 1. The molds were covered with plastic sheeting for 24 h to prevent evaporation loss. The specimens were then demolded and kept for water curing at room temperature until they were tested.

### 2.3. Testing Methods

The flowability of fresh grout is measured according to the flow time. To do this here, the freshly prepared grout is poured into a table-flow cone placed at the center of a base plate with a 300 mm diameter. After lifting the cone, the time taken by the grout to cover a diameter of 300 mm is observed, as T_30_, in seconds. The 28-day compressive strength levels of the grout mixes were examined with 50 mm cubic specimens.

**Figure 2:**
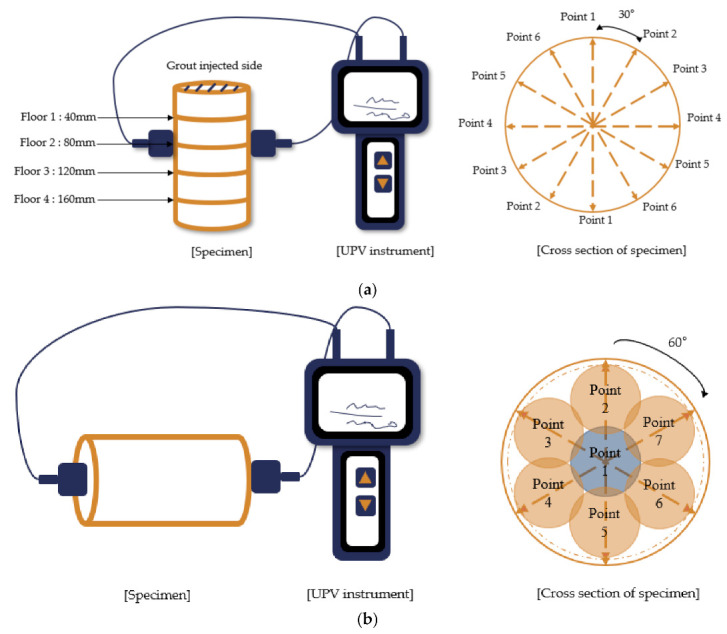
Setup for ultrasonic pulse velocity measurement: (**a**) Horizontal and (**b**) vertical.

In the PAAC case, all investigations were carried out on cylindrical specimens with 100 mm diameters and heights of 200 mm at 28 days of age. The compressive strength of the PAAC was measured as per the procedure in the ASTM C39 specification [19]. The UPV values were obtained on cylindrical specimens on both the horizontal and vertical section, as shown in Figure 2. Figure 2a shows the procedure and locations for the horizontal UPV values and Figure 2b shows the systematic approach used for the vertical UPV values. The ASTM C642 [20] guideline was followed to obtain the density, water absorption and total voids of the PAAC specimen. Three replicates were tested for each testing method.

## 3. Results and Discussion

### 3.1. Properties of the Grout

**Figure 3:**
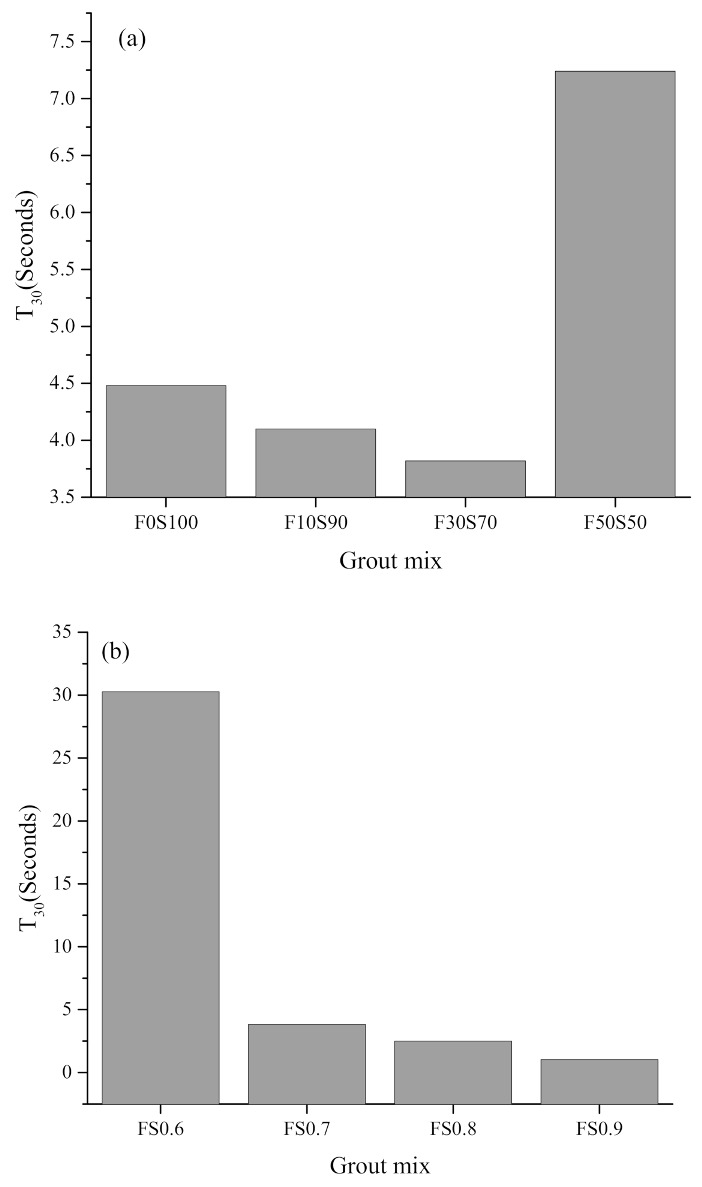
Flowability of alkali activated grout as observed by T_30_ time: (**a**) Series 1 and (**b**) series 2.

The flowability of the fresh grout mixes is shown in Figure 3. It should be noted that the CC grout was unable to cover the 300 mm diameter in the test. For the series 1 mixes, it can be seen that fly ash when incorporated up to 30% lowered the T30 flow time, increasing the flowability of the grout. However, when 50% fly ash was incorporated, the T30 flow time increased. The large particle size of fly ash enhances the interparticle friction causing an increase in the T30 flow time [17]. Furthermore, the different particle size between fly ash and slag promote particle interlocking and increases the T30 flow time. For the series 2 mixes, an increase in the solution-to-solid ratio from 0.6 to 0.9 had a positive effect on the ease of the grout flow, as the T30 flow time was reduced by nearly 96%. Nevertheless, all specimens of the alkali-activated grout had T30 times of less than 35 s. The CC grout is composed entirely of ordinary Portland cement, having angular particles which increase the interparticle friction. On the other hand, the comparatively spherical particle shape of the fly ash results in improved flowability [17]. This can also be due to the ball bearing effect of the fly ash, which reduces the interparticle friction, leading to an improved grout flow [21]. Moreover, the large specific surface area of the slag can influence the flow characteristics of the grout [17]. Another factor is a reduction in the amount of slag, which reduces the acceleration reaction between the precursor and the alkali-activating solution, leading to higher fresh flowability as compared to samples with higher amounts of slag [17,22]. These findings are in good agreement with the results in previous works [23,24,25]. Based on the T30 flow time values, the alkali-activated grouts can contribute to easing the workability during the placement process. Furthermore, regarding the setting time of alkali activated materials, detailed observations have already been reported in previous studies [17,26].

**Figure 4:**
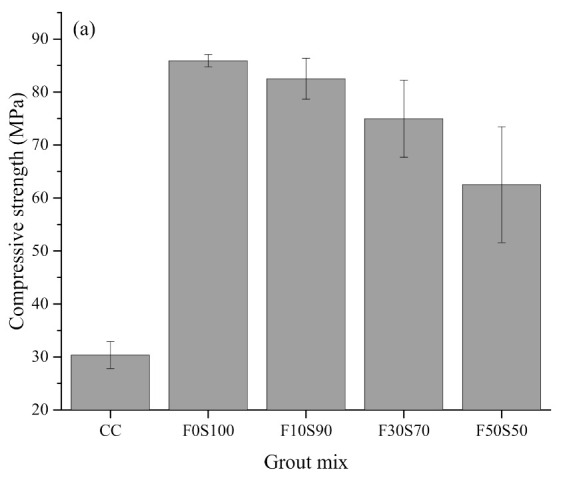

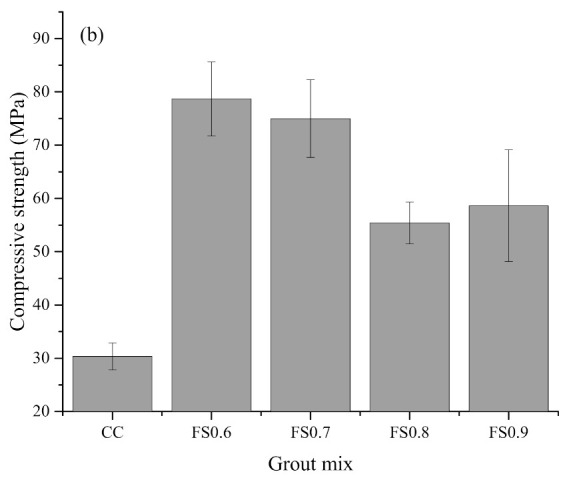
Compressive strength of grout mixes: (**a**) Series 1 and (**b**) series 2.

Figure 4 shows the 28-days compressive strength of the hardened grouts. It can be observed that both series of alkali-activated grouts exhibited higher compressive strength levels compared to those of the CC grout. The higher reactive capability [27,28] of the alkali-activated grout as compared to the CC grout can also lead to higher strength capabilities. The highest compressive strength was registered by the series 1F0S100 specimen. A decrease in the compressive strength was observed upon the incorporation of fly ash in the alkali-activated grout. Similarly, a decrease in the compressive strength upon an increase in the solution-to-binder ratio was observed in the series 2 specimens. For the series 1 specimens, the slag/fly ash content is an important factor governing the compressive strength of the grout. The reduction in the compressive strength at a higher ratio of fly ash in the slag mixes may be due to the formation of different types of reaction products [29]. It has been established that in a 100% slag alkali-activated system, C-A-S-H is the main reaction product [30,31], whereas upon the incorporation of fly ash, amorphous aluminosilicate gel generally forms [32,33]. Additionally, the sluggish reaction rate of fly ash as compared to slag is generally considered as a factor contributing to the reduction of the compressive strength [34]. Furthermore, a study by Samantasinghar and Singh [35] found that slag undergoes both alkali activation and hydration reactions. The nucleation effect of Ca^2+^ accelerates the hydration, thus contributing to the development of compressive strength [35,36]. This dual reactivity characteristic of slag can also play a role in enhancing the compressive strength. Another influential factor is the reactivity of the slag and the fly ash at room temperature. It has been reported that fly ash has a slower activation rate than slag at room temperature [26]. Alkali activation of fly ash usually requires curing at an elevated temperature to accelerate the rate of the reaction [37]. On the other hand, slag shows an accelerated activation rate at room temperature [26]. Because the present study focuses on curing alkali-activated grout at ambient temperatures, it is likely that this phenomenon can be responsible for the lower compressive strength upon an increase in the fly ash content. In the series 2 specimens, an increase in the solution content is usually the major factor behind the lower compressive strength. Earlier works indicated that an increase in the solution content usually decreases the alkali-activation process due to the higher consistency of the mixtures [25]. 

### 3.2. Compressive Strength of PAAC

**Figure 5:**
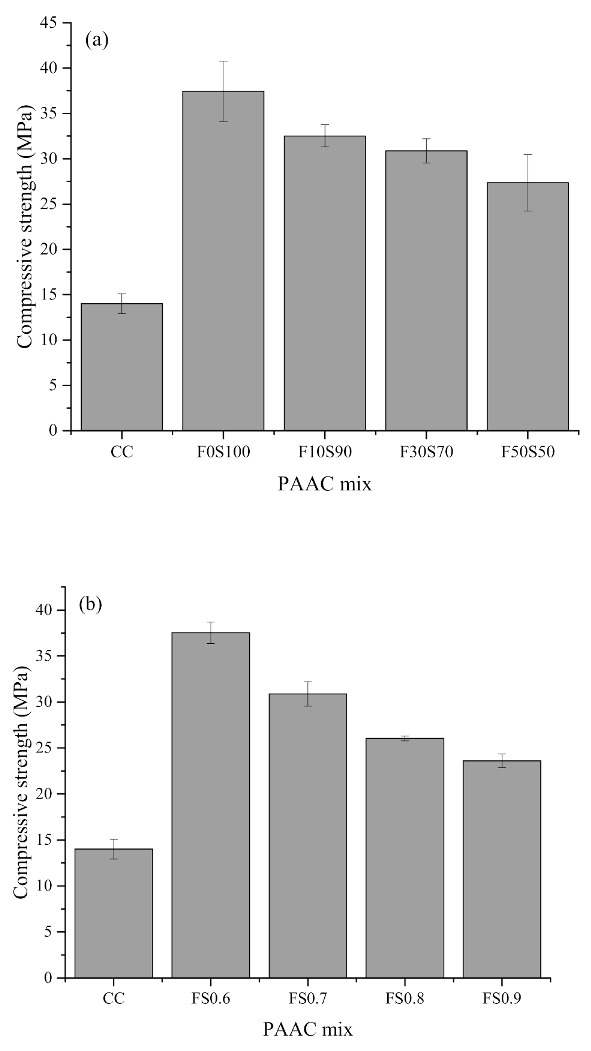
Compressive strength of preplaced aggregate concrete mixes: (**a**) Series 1 and (**b**) series 2. PAAC: Preplaced alkali-activated concrete.

Figure 5 shows the 28-days compressive strength of the PAAC specimens produced with different types of grout. It can be observed that the compressive strength of PAAC is fairly well correlated with the compressive strength of the grout. Specifically, the F0S100 specimen registered an increase of almost 166% in the compressive strength as compared to CC. Previous studies demonstrated that the compressive strength of PAAC is governed by the interlocking and strength of the coarse aggregate [38,39]. In the present study, the additional influence of the binding grout along with its flowability could be identified as factors influencing the compressive strength of the preplaced concrete.

The grout in this case is designed to ensure that the crushed stone aggregate remains in close contact to resist mechanical stress. The better compaction and filling capacity of the alkali-activated grout due to the greater flowability may have resulted in fewer voids between the coarse aggregates. The inherent flow characteristics of grout can directly influence the skeletal structure of PAAC, as insufficient penetration of the grout will lead to a honeycombed matrix. It can be seen that the compressive strength of PAAC represents about 50% of the grout strength. This phenomenon was also identified in a previous study [38] and was attributed to the point contact of coarse aggregates in the PAAC in that case. Conventional concrete is produced by mixing cement, water, sand and coarse aggregate. The mixing process ensures sufficient wrapping contact between the concrete-making materials and the formation of adhesive interfaces. Wrapping contact in this context implies the coating of cement mortar on the surface of coarse aggregate during the mixing process. On the other hand, in PAAC, the grout fills the void between the aggregate and provides point contact during mechanical loading. It should be noted that during the formation of PAAC, the dry coarse aggregate can absorb the liquid content from the grout and will influence the solution-to-solid ratio at the contact interface, strengthening these interfaces to some extent. Therefore, the solution-to-solid ratio also has a direct effect on the strength of the PAAC. However, it should be noted that a higher solution-to-solid ratio despite enhancing the flowability lowers the compressive strength of PAAC due to bleeding and weak adhesive interfaces [40].

### 3.3. Ultrasonic Pulse Velocity of PAAC

UPV is generally applied to identify cracks and voids in concrete and can provide an assessment of the quality of concrete. For the conventional concrete system based on ordinary Portland cement, it has been established that the compressive strength and UPV values are usually highly correlated. However, a study by Thunuguntla et al. found that the standard UPV values of conventional concrete may not reflect the quality of alkali-activated concrete [41]. The present study focuses on preplaced alkali-activated concrete, which has a quite different internal system from conventional concrete.

**Figure 6:**
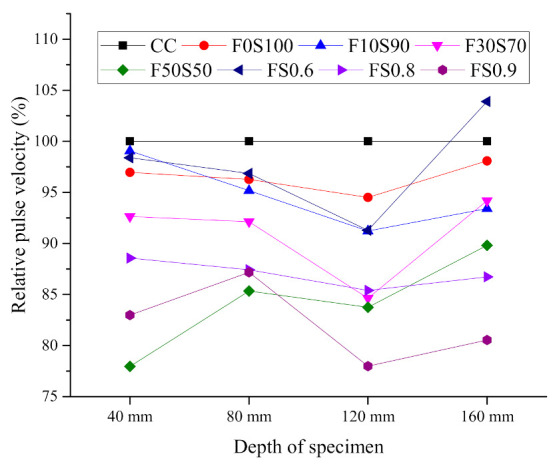
Filling capacity of grout mixes evaluated by relative pulse velocity at different depth of specimen.

The UPV values of the PAAC mixes are presented in Table 3. Figure 6 presents the filling capacity of the grout mixes at various specimen depths. The filling capacity was determined by evaluating the relative pulse velocity of each specimen at different depths. To provide a baseline for the measurements, the CC mix was considered as the ideal case scenario, with a relative pulse velocity of 100%. The successive relative pulse velocities of the PAAC mixes were calculated in relation to the CC mix. It can also be observed in Table 3 and Figure 6 that the increase in the fly ash content in the alkali-activated grout mixes resulted in lower UPV values. In the F0S100 mix, higher concentration of CaO-rich slag led to the formation of additional C-S-H and C-A-S-H gels through the alkali-activation reaction mechanism [42]. The production of these gels reduced the porosity and provided a denser medium, which is reflected in the UPV values. In the mixes with increased percentages of fly ash, the slower reaction rate of the fly ash reduced the formation of alkali-activated reaction gels, which resulted in decreased UPV values [43]. Additionally, it should be noted that FS0.6 registered the highest UPV at a depth of 160 mm for the alkali-activated grout samples. The increase in the solution-to-binder ratio did not necessarily result in a better filling capacity. The increase in the porosity due to the higher solution-to-binder ratio can be considered as a major factor behind such a phenomenon. The excess moisture in case of high solution to binder ratio gets leached out of the alkali-activated binder [44]. The loss of moisture results in a weak and porous structure thereby lowering the UPV values. 

The UPV measurements can also be indicative of voids between the aggregate and the paste which form in preplaced concrete. Voids between an aggregate and paste usually depend on the compacting ability of the infill grout used. However, in the present study, alkali-activated grout was used as an infill material. The flow of the series 1 binary blend of fly ash and slag is primarily dependent on the fly ash content. An increase in the fly ash content to 30% increases the flow characteristics but results in lower UPV values at all measurement positions. This can indicate that fly-ash-based infill grout has poor adhesive bonding with the aggregate, leading to the formation of voids in the preplaced concrete skeletal system. 

The UPV positioning was also investigated in the present study with regard to horizontal and vertical measurements. The UPV values with regard to positioning are presented in Table 3. A distinct difference can be observed for UPV values based on horizontal and vertical positions. The UPV values can provide a background on which to base comments about the filling capabilities of various grouts at the investigated locations. For the vertical measurements here, it was found that the values obtained on the side location are considerably lower than those on the center location for the same specimen. This may be due to the close gap between the surface of the mold and the coarse aggregate, which did not allow efficient wrapping by the grout.

### 3.4. Density, Water Absorption, and Porosity of PAAC

**Figure 7:**
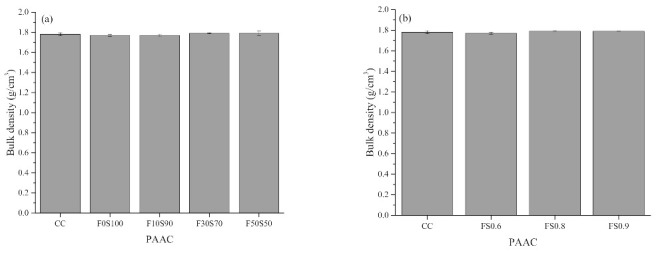
Bulk density of preplaced aggregate concrete mixes: (**a**) Series 1 and (**b**) series 2.

**Figure 8:**
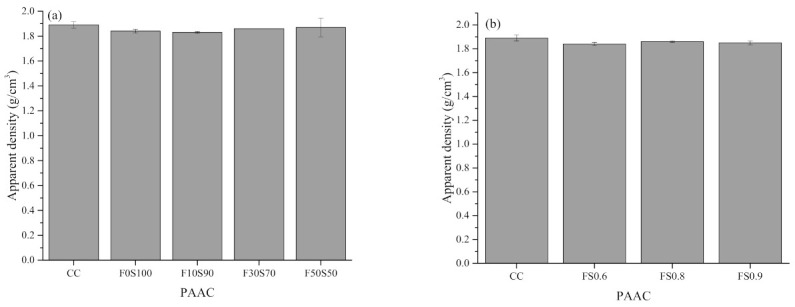
Apparent density of preplaced aggregate concrete mixes: (**a**) Series 1 and (**b**) series 2.

**Figure 9:**
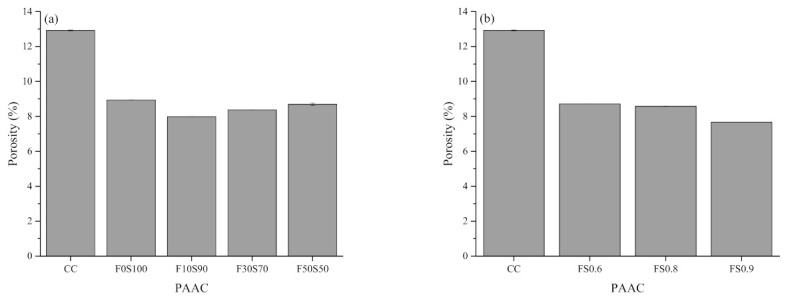
Porosity of preplaced aggregate concrete mixes measured by the total void test: (**a**) Series 1 and (**b**) series 2.

**Figure 10:**
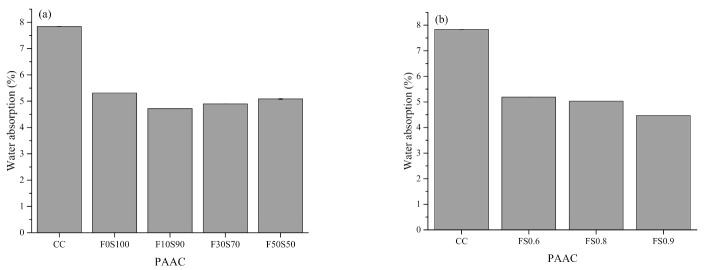
Water absorption of preplaced aggregate concrete mixes: (**a**) Series 1 and (**b**) series 2.

Figure 7, Figure 8, Figure 9 and Figure 10 summarize the bulk density, apparent density, water absorption by immersion, water absorption after boiling, and total voids of the PAAC mixes. It can be observed that the use of alkali-activated grout resulted in similar bulk density, lower apparent density, water absorption, and porosity outcomes in PAAC. The bulk density considers both the solid volume and closed and open voids, whereas the apparent density excludes open voids. The measurements show that the use of alkali-activated grout in PAAC lowers the apparent density, which suggests a difference between the filling mechanisms by CC and the alkali-activated grout. However, it should be noted that the incorporation of fly ash in amounts exceeding 10% resulted in an increase in the apparent density for PAAC.

Less water absorption and fewer voids were also found in the PAAC mixes. The mutual relationship between the fewer porosity and the lower water absorption is the primary factor for this result. The better compaction and higher flowability of the alkali-activated grout compared to those of the conventional cement grout may have resulted in a dense structure. Specimens with more fly ash showed lower water absorption, contrary to the results obtained in previous studies of traditionally placed alkali-activated concrete [45,46,47]. This difference can be explained by the type of placement used and the preconditioning of the specimen. As per ASTM C642 [20], the PAAC specimens were oven-dried at 110 °C for 24 h. In the F0S100 mix, desiccation and dehydration of the C-A-S-H product can lead to higher porosity and greater water absorption. These effects are negated in fly ash mixes, as water present in the system becomes similar to zeolitic water (physically bound) and does not damage the microstructure as does the chemically bounded water in the slag mix [48]. A similar observation was reported by Ismail et al. when studying the influence of fly ash in alkali-activated slag mixes [49]. Additionally, in this study the wrapping of the grout around the coarse aggregate materials can be affected by dehydration and can create weak zones or microcracks, which can act as trap pockets for water during the immersion step, which in turn can lead to higher water absorption and porosity rates.

Two other interesting aspects observed in the present study are the lowering of the water absorption and the lower porosity in the PAAC mixes with higher solution-to-solid ratios. In the case of PAAC, the paste volume fraction is the key factor influencing the porosity. The higher flow characteristics of grout mixes produced with higher solution-to-solid ratios have been discussed in this study. During the placement in the formwork of the alkali-activated grout with a higher solution-to-solid ratio, the surface area of the water film being absorbed by the aggregates increases, which increases the surface area of the wrapping zone between the aggregate and the grout. This phenomenon reduces the paste volume required to cover the surface of the aggregate and reduces the actual solution-to-solid ratio, which results in the formation of a discontinued and disconnected pore network, thereby reducing water absorption. Similar observations of the effects of the paste volume were reported by Piasta and Zarzycki [50].

### 3.5. Discussion

The production of preplaced concrete by utilizing alkali-activated grout as an infill material presented substantial new details regarding the performance of PAAC and the scope of the alkali-activating grout. The alkali-activated grouts registered better fresh properties than ordinary Portland cement grout. Additionally, the incorporation of fly ash into the alkali-activated slag resulted in increased flow characteristics. The reduced flow time was indicative of the better compacting ability of the grout, which can lower the energy required during the infill action for preplaced concrete. However, the incorporation of fly ash in alkali-activated slag resulted in decreased compressive strength of the grout. The slow alkali-activation rate of the fly ash at ambient temperatures was a major factor in the decline of the compressive strength of the grout in this study.

**Figure 11:**
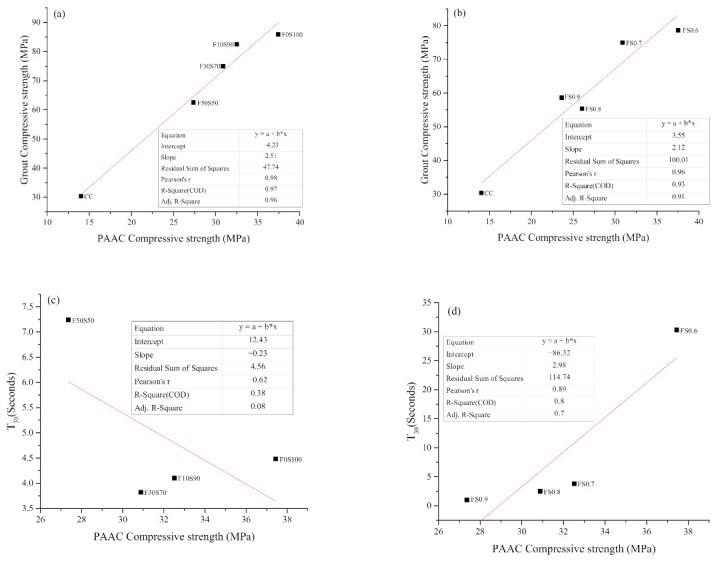
Correlation between grout and PAAC compressive strength: (**a**) Series 1 and (**b**) series 2, and, correlation between flowability of grout and PAAC compressive strength: (**c**) Series 1 and (**d**) series 2.

Two different relationships were found to exist between the properties of the grout and compressive strength of PAAC. Figure 11a shows the relationship between the compressive strength of the grout and the compressive strength of PAAC. A linear relationship with a correlation factor (R-square) of 0.976 was observed. This relationship indicates that alkali-activated grout as an infill material promotes the mechanical properties of PAAC to a greater degree than ordinary Portland cement grout. Moreover, the introduction of fly ash into alkali-activated slag grout lowered the compressive strength of PAAC. As shown in Figure 11b, variation of the solution-to-binder ratio followed the well-established inverse relationship between the compressive strength and the solution content [40]. In the present study, a correlation factor (R-square) of 0.93 was observed. However, it must be noted that the PAAC fabricated with a solution-to-binder ratio of 0.9 registered higher compressive strength than the CC mix. This indicates a higher strength gain and better adhesion characteristics of the alkali-activated grout as compared to CC grout. The second relationship observed in the present study is between the flowability of grout and the compressive strength of PAAC. Figure 11a shows that a scattered relationship exists between grout produced with different amounts of fly ash and the compressive strength of PAAC. On the other hand, as shown in Figure 11b, an inverse correlation is observed for grout produced with varying solution-to-binder ratios and the compressive strength of PAAC. From these two relationships, it can be stated that the compressive strength of grout plays a much more defining role with regard to the mechanical performance of PAAC as compared to the grout flow characteristics (Figure 11c,d). However, it must be noted that the flow characteristics in fact play have a secondary role in that they warp the aggregate to create a cohesive product. Overall, it can be concluded that the designed PAAC can overcome the shortcoming of the low compressive strength of CC-mix-based preplaced concrete.

The approach here pertaining to the development of an alkali-activated grout for the fabrication of PAAC indicates that the quantity of fly ash should be kept below 50% to obtain favorable flowability and compressive strength characteristics of grout. In addition, the solution-to-binder ratio should be designed with the utmost care to obtain the preferred flow characteristics without compromising the mechanical performance of PAAC. However, future studies are required to understand the impact of temperature, molarity of alkali activating solution and aggregate size on properties of PAAC. In case of temperature, it has been pointed in various studies that higher temperature results in higher gain in compressive strength in a shorter duration of time due to enhanced polycondensation [51,52]. For molarity of alkali activating solution, it has been widely reported that higher molarity results in enhanced dissolution of precursor which results in higher compressive strength [53,54]. Furthermore, the molarity of solution also affects the fresh property of alkali activated grout [52]. The higher molarity of alkali activating solution results in decreased initial and final setting time [55]. The aggregate size in manufacturing of PAAC depends upon multiple factors. It is usually recommended to avoid aggregate smaller than 20 mm size as they restrict the grout injection pipes and impedes the grout flow [56]. However, the minimum size of coarse aggregate depends on the grout mix type and method of placing the grout to produce PAAC.

## 4. Conclusions

The present study investigated the effects of alkali-activated grout on the characteristics of preplaced aggregate concrete. The investigations included experimental designs to examine the flow characteristics and compressive strength of the grout as well as tests of the compressive strength, ultrasonic pulse velocity, density, water absorption, and total voids of the preplaced concrete. The main conclusions drawn from the work are as follows.

(1) The flowability results showed that alkali-activated grout demonstrated increased flow time values as compared to ordinary Portland cement grout. The incorporation of fly ash up to 30% in alkali-activated grout enhanced the flow characteristics.
(2) The compressive strength of preplaced concrete was significantly improved with alkali-activated grout as a filling agent. However, the incorporation of fly ash resulted in decreased mechanical performance. The slower reaction rate and different reaction products were considered as the primary factors.
(3) Location-based UPV measurements showed that concrete the surface close to the mold surface has a weak skeleton as compared to that at the center location. For PAAC, a good correlation between the UPV values and compressive strength was observed for increased amounts of fly ash. However, an inverse relationship was found to exist between the UPV and total void values.
(4) As the content of fly ash was increased, the water absorption and porosity decreased. The specimen preconditioning and the primary reaction compounds were the main contributing factors. The changes in the surface area of grout wrapping and water absorption by the aggregate resulted in lower porosity and water absorption rates for the alkali-activated PAAC specimens fabricated with higher solution-to-solid ratios.
(5) A linear relationship between the compressive strength of grout and PAAC was observed. The raw materials mix design along with the solution-to binder-ratio were recognized as critical parameters to produce alkali-activated grout for fabricating PAAC.

## Figures and Tables

**Table 1:** Chemical composition of slag and fly ash.

Composition (%)	Slag	Fly Ash
SiO_2_	36.5	53.5
CaO	36.6	5.1
Al_2_O_3_	16.3	26.8
Fe_2_O_3_	0.2	7.0
SO_3_	3.04	1.0
MgO	5.9	1.5
P_2_O_5_	0.1	0.7
K_2_O	0.5	1.5
Na_2_O	-	0.9
TiO_2_	-	1.4
SrO	-	0.2
BaO	-	0.2

**Table 2:** Mix proportions of grout.

Mix Series	Mix Nomenclature	Liquid/Binder	Cement	Slag	Fly Ash
Series 1	CC	0.5	1	0	0
	F0S100	0.7	0	1	0
	F10S90	0.7	0	0.9	0.1
	F30S70 *	0.7	0	0.7	0.3
	F50S50	0.7	0	0.5	0.5
Series 2	FS0.6	0.6	0	0.7	0.3
	FS0.7 *	0.7	0	0.7	0.3
	FS0.8	0.8	0	0.7	0.3
	FS0.9	0.9	0	0.7	0.3

* F30S70 and FS0.7 are identical mixes in the two series.

**Table 3:** Ultrasonic pulse velocity (UPV) values of preplaced aggregate concrete mixes.

Position	Location (mm)	CC (Km/s)	F0S100 (Km/s)	F10S90 (Km/s)	F30S70 (Km/s)	F50S50 (Km/s)	FS0.6 (Km/s)	FS0.8 (Km/s)	FS0.9 (Km/s)
Horizontal	40	3.45 ± 0.07	3.34 ± 0.28	3.49 ± 0.2	3.19 ± 0.17	2.69 ± 0.08	3.39 ± 0.12	3.05 ± 0.17	2.86 ± 0.06
	80	3.38 ± 0.21	3.26 ± 0.14	3.22 ± 0.15	3.12 ± 0.21	2.89 ± 0.08	3.28 ± 0.05	2.96 ± 0.15	2.95 ± 0.09
	120	3.48 ± 0.08	3.29 ± 0.11	3.17 ± 0.2	2.94 ± 0.15	2.91 ± 0.18	3.17 ± 0.09	2.97 ± 0.07	2.71 ± 0.29
	160	3.25 ± 0.22	3.19 ± 0.19	3.03 ± 0.14	3.06 ± 0.14	2.92 ± 0.1	3.38 ± 0.69	2.82 ± 0.21	2.62 ± 0.05
Vertical	Side	3.16 ± 0.07	2.65 ± 0.13	2.76 ± 0.21	2.67 ± 0.07	2.85 ± 0.33	3.14 ± 0.22	2.51 ± 0.08	2.11 ± 0.01
	Center	3.35 ± 0.09	2.94 ± 0.25	3.05 ± 0.42	2.60 ± 0.36	3.07 ± 0.27	3.35 ± 0.26	2.68 ± 0.18	2.40 ± 0.11

## Data Availability

The data presented in this study are available on request from the corresponding author.
